# Typologies of women’s abortion trajectories in Burkina Faso: findings from a qualitative study

**DOI:** 10.1186/s12978-022-01526-3

**Published:** 2022-11-28

**Authors:** Fiacre Bazié, Haley L. Thomas, Meagan E. Byrne, Boukary Kindo, Suzanne O. Bell, Caroline Moreau

**Affiliations:** 1 Institut Supérieur des Sciences de la Population (ISSP), Université Joseph Ki-Zerbo, Ouagadougou, Burkina Faso; 2 Department of Population, Family and Reproductive Health, Johns Hopkins Bloomberg School of Public Health, Baltimore, MD USA; 3 Soins Primaires et Prévention, CESP Centre for Research in Epidemiology and Population Health, U1018, Inserm, 94800 Villejuif, France

**Keywords:** Burkina Faso, Abortion, Pregnancy termination, Reproductive health, Qualitative research

## Abstract

**Background:**

Abortion is a legally restricted, yet common reproductive life event among women in Burkina Faso; however, women’s abortion experiences vary greatly depending on their social and economic capital, partner involvement, and level of knowledge. We sought to classify women’s abortion care-seeking experiences across the life course and social conditions into typologies using qualitative data.

**Methods:**

An initial quantitative survey among a nationally representative sample of women ages 15–49 years collected information on women’s induced abortion experiences. Women who reported an abortion in the last 10 years were asked if they would agree to participate in a subsequent in-depth interview (IDI) to learn more about their abortion experience. Twenty-five women, identified via purposive sampling, completed an IDI. Using a process of typology construction, we identified attributes of each woman’s condition at the time of her abortion and aspects of her abortion experience, created a matrix of attributes and cases, and identified emerging relationships. Three types were identified through this process.

**Results:**

Twenty-three IDIs were analyzed, and women’s abortion experiences were grouped into three types: abortion to delay childbearing in adolescence; abortion to space childbirth among women in union; abortion to avoid childbearing among single mothers. Two cases were identified as outliers. Cases were grouped based on the context of their pregnancy, the reason for the abortion and involved decision-makers, and their patterns of care-seeking, including methods and sources used.

**Conclusion:**

Structural inequities related to gender and wealth were prominent forces shaping women’s abortion experiences. Comprehensive sexuality education coupled with community-based interventions to promote gender-equitable relationships and address social stigma related to women’s sexuality could reduce reproductive coercion and unintended pregnancies.

## Background

In Burkina Faso, induced abortion is legally restricted and yet a common reproductive life event, with a recent estimate of 23 (95% confidence interval 16–30) abortions per 1000 women age 15–49 in 2020 [1]. The majority are performed using non-recommended methods by untrained providers [1, 2], and many women suffer serious complications [2, 3]. A qualitative study of women’s abortion health-seeking trajectories in Ouagadougou corroborates these findings. The study describes a range of unsafe and ineffective practices, from taking bitter drinks to ingesting different pharmaceutical products, leading to frequent complications [4]. For many, health centers were seen as a last resort rather than an entry point of care [4]. Cost is a major barrier to accessing these services. In 2015 in Ouagadougou, the average cost of $89 for an abortion [5], corresponded to 15% of the gross domestic product per capita at the time [5]. The inability to receive treatment for abortion complications can have severe repercussions, as abortion complications contribute to 10–18% of maternal mortality, which at 330 per 100,000 births remains well above SDG 2030 goals [6, 7].

While the frequency of unsafe abortion is well-documented across settings with legal restrictions [8], women’s abortion experiences greatly depend on their social and economic capital which facilitates access to medical advice and services [9–12]. Recent research in Nigeria and Côte d’Ivoire—two neighboring countries with similarly restrictive abortion laws—reveals stark social disparities in abortion safety by wealth, education, and residence [13, 14], which are amplified by social disparities in access to quality postabortion care to address complications [15]. Qualitative research in Burkina Faso highlights the importance of women’s social networks as sources of information and support to navigate abortion decisions and access to care [16]. Research in urban Zambia also points to the critical role of male partners as facilitators or barriers to safe abortion care [18]. Findings suggest that men’s financial support and ability to mobilize resources and their social networks greatly influence women’s abortion care pathways [18]. These observations are confirmed in a recent quantitative study in Nigeria and Côte d’Ivoire, with 66–73% lower odds of unsafe abortion when partners are involved [19].

The availability of misoprostol, a safe and effective abortion drug [20, 21], outside of the healthcare system is rapidly changing the abortion landscape, offering women the potential to safely self-manage their abortion [22]. Self-managed abortion using misoprostol has greatly reduced abortion-related morbidity and mortality in South America [23–25], and is gaining traction globally, although most women in West Africa are unaware of this safe option [1, 26, 27]. Qualitative research in urban Burkina Faso shows that some women rely on misoprostol to self-induce, but points to social inequities in access as many women are not economically or socially empowered to access misoprostol [4, 28].

Given the changing abortion landscape in a context of growing social aspirations to have fewer children in Burkina Faso, there is a need to better understand the circumstances in which women decide to terminate a pregnancy and how they navigate access to abortion care according to their socioeconomic conditions and their partnerships. Using qualitative data collected from women who reported a recent abortion experience, we specifically aim to identify typologies that capture different experiences from decision-making to care-seeking conditions across women’s life course and social conditions in Burkina Faso.

## Methods

This study is part of a larger mixed-methods study conducted in 2020–2021 by Performance Monitoring for Action (PMA), a multi-country research platform monitoring reproductive health and family planning indicators through annual population-based surveys. The study was designed to estimate the incidence and safety of abortion in Burkina Faso and to understand the intersecting social, economic, and institutional factors influencing women’s pathways to abortion care (ISSP 2021).

An initial quantitative survey among a nationally representative sample of WRA (ages 15–49) collected information on women’s induced abortion experiences. The survey asked women if they had ever done anything to intentionally end a pregnancy or if they had ever done anything to bring back a late period when they were worried about being pregnant; we categorized both as abortion. Those who reported an abortion (any of the previous described experiences) in the last 10 years were asked about methods and sources of abortion care, complications, and quality of care. They were also asked if they agreed to participate in a subsequent qualitative study to learn more about their experience.

This qualitative analysis focuses on a purposive sample of 25 women who reported an abortion in the quantitative survey, consented to be recontacted, and who represented a diversity of experiences according to age, socioeconomic and marital status at the time of the abortion, area of residence, and abortion care trajectory. Women were contacted via phone by a trained interviewer who followed an IRB approved script to verify prior survey participation, to confirm interest in participating in the qualitative follow-up study and to set a time and place of the women’s choice to conduct face-to-face in-depth interviews (IDIs). The interviewers were 6 female research assistants with experience in qualitative research and fluency in French and local languages. They participated in a three-day interviewer training to ensure comprehension of the IDI guide and the inclusion of appropriate questions and probes.

Interviewers obtained women’s verbal informed consent to participate before starting the IDI, in French or a preferred local language, which was audio-recorded with participant consent. The interviews lasted 60 min on average. The full study protocol and all study materials were approved by the Johns Hopkins Bloomberg School of Public Health Institutional Review Board and the Comité d’Ethique pour la Recherche en Santé of the Ministère de la Santé et de l’Hygiène Publique et Ministère de l’Enseignement Supérieur, de la Recherche Scientifique et de l’Innovation in Burkina Faso.

The IDI guide, which was pilot tested during the training, solicited women’s perspectives on a range of topics, including the practice of abortion in their community, their own abortion experience–from the circumstances surrounding the decision to terminate the pregnancy to abortion care and post-abortion complications–and their recommendations to reduce unsafe abortion in their community. Women were probed about the role of others (particularly their partner) in relation to their decision to terminate and access abortion care. They were asked to describe how they chose the sources of care and methods used, their pathways to abortion, and their interactions with providers and other sources of support during their abortion trajectory. Additionally, women were probed about their physical health and emotional reactions during the abortion process and the quality of care they received. Before the start of the interview, participants provided basic sociodemographic information about their age, level of education, employment, parity, and marital status, as well as their age and partnership situation at the time of the abortion.

The authors, a team of five researchers (two at ISSP and three at JHSPH), conducted the data analyses in French. IDIs were first transcribed verbatim if conducted in French or directly translated and transcribed into French if conducted in another language, with random checks by the ISSP research team to assess the quality of the transcriptions. The researchers began with a process of data familiarization by conducting a close reading followed by writing a structured summary of each transcript. Next, the team undertook a more focused analytical exploration to identify typologies of abortion trajectories, based on abortion pathways and the socio-economic and familial conditions, which informed women’s abortion-related decisions, their health, and their social safety. We organized and analyzed the data with the assistance of Microsoft Excel (v16.48).

Specifically, the research team followed a four-step process for typology construction [29], first identifying the relevant attributes, or dimensions, of women’s abortion trajectories. The attributes were informed by the interview guide, reflecting previous literature on the role of partner involvement, social network involvement, and socioeconomic status, and further refined by the major themes emerging from analysis of the detailed summaries of each interview, including reproductive life course and family arrangements. In the second step, the team created a matrix crossing each IDI with the identified attributes that fell into two categories: woman’s condition at the time of abortion (socioeconomic background, reason(s) for abortion, partner and family interactions, social network, and social stigma) and aspects of her abortion experience (information sources, sources and methods of abortion, complications). The team then grouped IDIs to identify “empirical regularities” across all interviews based on commonalities of attributes. This process began with two separate analysts creating initial groupings of types based on regularities identified. This led into the third step in which groups of IDIs were analyzed for meaningful relationships and additional attributes were explored. Any discordance between groupings created by the analysts was discussed among the wider research team to reach consensus. Finally, we analyzed the three emerging types, reflecting common combinations of attributes relating to women’s abortion trajectories.

## Results

In total, 25 women consented and completed an IDI; however, two women who reported an incomplete abortion that resulted in a birth, were dropped from this analysis. Among the remaining 23 participants 21 had their abortion in the three years prior to the quantitative survey and 2 had their abortion within 5–10 years. At the time of the abortion, participants were aged between 15 and 42 years, 16 were in short-term or first partnerships, and 11 had children (Table 1). Ten women did not disclose their pregnancy to their partner or did not involve them in the abortion process. Twelve women sought care from non-clinical sources, including friends, street vendors, and pharmacies, and eight described abortion complications, six of whom received postabortion care.

**Table 1:** Respondents’ characteristics

	N = 23
**Age at the time of the abortion**	
≤ 19	10
20–29	10
30 +	3
**Relationship at the time of the abortion**	
First relationship/partnership	2
Casual/short-term relationship	14
Long-term relationship/married	7
**Partner involvement**	
Informed of pregnancy	17
Involved in abortion decisions/process	13
**Non-clinical source of abortion care**	10^a^
** Reported an abortion complication**	8

^a^Two of these 10 women could not determine if their provider was a medical professional

Observing the similarities and differences in the conditions underlying abortion decisions and healthcare-seeking experiences, we identified three types of abortion pathways, primarily informed by attributes connected to women’s life course in terms of age and partnership status at the time of the abortion and by their social capital and resources. Below we describe each typology, highlighting the context in which the pregnancy termination occurred, the reasons for the abortion, the people involved in the decision-making, and finally the sources and methods used for the abortion.

### First typology: Abortion to delay childbearing in adolescence

#### Context of pregnancy

A total of 11 adolescents, between 15 and 19 years of age at the time of the abortion, comprise this group of abortion experiences at the start of their reproductive lives.

In this early stage of the reproductive life course, adolescents had little sexual experience when they became pregnant during their first partnership, lacking knowledge and/or agency to prevent pregnancy. In particular, many were uninformed about contraception, leading to unprotected sex or ineffective means of protection, while others recognized the risk of pregnancy but were unable to negotiate protection with their partner. Lack of reproductive knowledge also resulted in delayed pregnancy recognition–often by several months–even when overt signs were visible.

The abortion I had to have […] at the time I was in class 6 (middle school), I was living with my older sister at the time. Meanwhile, my periods were not coming, and as it was my first pregnancy, I did not have any experience, I did not know it was a pregnancy; each time I would go to PE (sports), I would feel pain in the lower part of my belly, so at home, I would take some Ibuprofen to relieve the pain and when going back to PE, I felt pain again in my lower belly so I did not understand […] listen, he was my first sexual partner, and when we did it, it was the first time and it was a pregnancy at the same time.

(15 years old, no children, secondary level education, living in an urban at the time of abortion).

The pregnancy happened… actually, I don’t know how it happened (laugh), that I don’t know, but I knew I was pregnant, that’s it. I went to take the test, and knew I was pregnant; because at the time, I can say we were having unprotected sex, it’s because we didn’t use protection that the pregnancy came in. It’s true that in the beginning, he would always say, “let’s do the withdrawal method, let’s do coitus interruptus, but in the end, with his coitus there, the pregnancy came in (smile).

(17 years old, no children, secondary level education, living in an urban area at time of abortion).

I was two months late. Two months, my periods didn’t come and as often, they do come, they weren’t coming so I didn’t know. I said I would wait for the second month and see, and when it was two months, I went to take a pregnancy test, and I saw I was pregnant […]

(16 years old, no children, secondary level education, living in an urban area at time of abortion).

#### Abortion decision

When these adolescent girls became pregnant, they were unmarried, living with their family, mostly in urban areas, and in school. Two main reasons drove their decisions to terminate their pregnancy: fear of family sanctions and partner disapproval.

Social stigmatization and fear of family reprisal were prominent reasons for secretly terminating an early pregnancy. Pregnancy among unmarried girls still living with their family is considered a “dishonor” for the family, bringing shame in many communities in Burkina Faso. Adolescents feared being banned from their homes, thereby losing social and economic support. One girl described her sister’s experience of becoming “a stranger to the family” when she became pregnant and was banned from the household. This experience informed her decision to terminate her pregnancy to avoid the same predicament. Several young respondents shared the same fears, hiding their pregnancy to avoid sanctions.

(silence) I was scared because if my father learned I was pregnant, he would tell me to find a place to go and to know who the father was. But since he [my boyfriend] said it was not his pregnancy, I got scared. If my father told to me to find a place to go, where would I go? That’s why, I was scared.

(18 years old, no children, secondary level education, living in an urban area at time of abortion).

Partners’ reaction to the pregnancy also strongly influenced adolescent girls’ decisions. Most of their partners were young, unwilling to give up their educational and professional aspirations, and unable to economically support a family at this early stage. In some cases, the partner’s decision to terminate the pregnancy aligned with the young woman’s desire to postpone childbearing to continue studying and find economic stability before starting a family.

[…] the final decision came from both of us. The fact that [he] convinced me as he said ‘see, your parents are opposed to it, I like you and I want you to continue studying so we can go far and have kids; and see my situation right now, I don’t have a job, I get by. How can we raise this child if he were to be born?’ I could see how everything he said was true; I could see that this child would somehow really stop my life. That’s why I finally said yes. I said OK, let’s find a solution, let’s do it.

(17 years old, no children, secondary level education, living in an urban area at time of abortion).

In other cases, however, girls felt coerced by their partners to continue or terminate the pregnancy. Some were pressured to have an abortion because their partners denied responsibility or economic support, as described below in Sect. 2.3.

One young woman kept the pregnancy secret from her partner out of fear of being coerced into having a child when she was not ready to start a family.

#### Care-seeking

Few adolescents knew about sources and methods of abortion, and therefore, relied on their partner or another close confidante (friend or family member) to access care. These actors played a critical role in informing healthcare decisions by mobilizing their social networks and economic resources, or sharing their personal experiences. Their role was amplified by the need to maintain confidentiality which restricted women’s ability to seek other sources of information, often at the expense of their physical safety if the advice they received led them to obtain unsafe abortions.

Partners could play a key role in abortion care pathways, depending on their sense of responsibility. Most partners assumed the financial cost, some were involved in method acquisition, and a few accompanied their partners during the procedure. In most cases, partner involvement opened the doors to safer abortions, as they could activate their network and mobilize economic resources to cover expenses. This was the case for one adolescent girl who said that she did not know how to induce the abortion, so her partner provided her with the tablets (misoprostol):

He [my boyfriend] said he cannot, he doesn’t want me to keep it. So, to do everything I can to remove this. I said I don’t know how to remove it, it’s dangerous, I can leave [die] with that. So, he said that no, he will give me a medicine, that I will do it. He came to give me the medicine, like a pill, I took it and the next day, I felt pain in my lower belly. So, after that, I started to bleed. So, that’s it.

(17 years old, no children, primary level education, living in an urban area at time of abortion).

While partners could facilitate access to recommended pills or clinical care, they sometimes also compromised the girl’s ability to make their own healthcare decisions, such as in the case of one adolescent girl who was told “to prepare [herself] that the abortion will take place next Tuesday” and was driven to the facility by a partner who pressured her to have the abortion.

No, he [my boyfriend] did not let me talk to the guy [the abortion provider]; when we left, he simply told me we would remove it, he drove me on the back of his motorcycle, we took off, he talked with the guy and he let me in, that’s all, when that guy was finished with his work, we left.

(16 years old, no children, primary level education, living in an urban area at time of abortion).

In the absence of partner support, many adolescent girls relied on their best friend or a family member for their healthcare decisions, who made a range of recommendations from traditional methods to clinical care, involving a “doctor” in a “hospital” setting.

I explained my situation to my girlfriend, she said I should crush “Kossafandé”, drink and purge, and so I did that first, but it didn’t work, the pregnancy didn’t come out; then, I took some pills, it didn’t work, the pregnancy didn’t come out.

(15 years old, no children, secondary level education, living in an urban at the time of abortion).

One young respondent went to a doctor recommended by her close friend, as she was scared that if she discussed her abortion decision with her partner, he would want her to keep the pregnancy:

She [my friend] told me that to go to a doctor there [...] that the doctor there performs abortions. That she, she knows him, that he had performed an abortion on a girl, so we left together to the doctor there. We explained to him now, he told us that we had to leave and then come back afterwards and he told us the price.

(16 years old, no children, secondary level education, living in an urban area at time of abortion).

Regardless of methods and sources, many young women described abortion as a painful process, with bleeding lasting several days. Half of adolescents described complications requiring additional treatment. Delayed care due to late pregnancy recognition or lack of funds, coupled with substandard or inappropriate care, likely contribute to abortion failure and complications, which require advanced treatment at an additional financial and social cost. This was the case for a 19-year-old girl whose abortion nearly “cost her life”:

A lot! A lot of blood. Meanwhile, I was exhausted, and with each drop that fell, even before the drop falls, you cry. You cry even before the drop has started falling, and it’s as if there were pieces of your own flesh coming out. Each time you bleed, you cry, you cry… it wasn’t easy.

(19 years old, no children, secondary level education, living in an urban area at time of abortion).

She was hospitalized for dilation and evacuation and describes complications after receiving misoprostol from a doctor, which required a second treatment of misoprostol at an additional cost of 70,000 CFA (US$114), which she paid instead of her school fees.

Hmm… when this kind of situation happens to you… when I tried the first time and it did not come out, I was so panicked, I even said if I had to die with the pregnancy, it didn’t matter. So, I was really determined to remove it, especially that my boyfriend reacted as he did. I was so mad at him, and there you go. I even said if… whatever happens. When it happens, you don’t think of what will happen next. It’s risky, really, but…

(19 years old, no children, secondary level education, living in an urban area at time of abortion).

This was similar to another adolescent who received a medical abortion in a healthcare facility and ended up unconscious after bleeding for several days.

### Second typology: Abortion to space childbirth among women in union

Five married women, who were between the ages of 20 and 33 and had at least one young child at the time of the abortion, comprised this second type of abortion pathways in this study.

#### Context of pregnancy

In this later stage of the reproductive life course, women were raising a young family at the time of the abortion and many were engaged in income-generating activities. All became pregnant in the postpartum period while breastfeeding. This was the case for one woman who had a 7-month-old child when she became pregnant. She warned her husband, who had just returned from the gold mines, about the risk of pregnancy, but to no avail. Her husband coerced her into having sexual intercourse during her “ovulation period,” leading to the pregnancy.

I made him understand that it wasn’t the right time to have intercourse that day, but despite this, he forced me to have intercourse with me and he went back to the site. In the months that followed, my periods wouldn’t come, that’s when I knew I was pregnant.

(32 years old, 2 children, primary level education, living in a rural area at time of abortion).

#### Abortion decision

For all these women, social stigma related to short pregnancy intervals, as well as health and economic concerns, informed their decisions to terminate their pregnancy. They generally made this decision jointly with their partners, with the one exception of a woman who was in an undesired marriage and feared reproductive coercion in which her husband would force her to keep the pregnancy if he learned she was pregnant.

Shame over “uncontrolled” fertility was a common theme for women who became pregnant in the postpartum period, as it constitutes a transgression from traditional norms in Burkina Faso prescribing sexual abstinence during this period to ensure “appropriate” birth spacing. Such traditional practices are gradually being abandoned as couples resume sexual activity earlier in the postpartum period, which leaves women at greater risk of unintended pregnancy in the absence of contraceptive use. Faced with the prospect of a short birth interval, some couples agreed to terminate the pregnancy to avoid social shaming of having children too close together. This is reflected in one respondent’s story when she reported that her husband seemed as sad as she was when she informed him that she was pregnant. This sadness was related to the community’s view and judgment that they were having births too close together as she had a baby that “was still at the breast.”

Honestly, he was as sad as I was. His sadness was more linked to people’s perceptions of him, he was wondering what people would think of him, how they would see him, it was a source of shame for him (laugh).

(32 years old, 2 children, primary level education, living in a rural area at time of abortion).

Social norms to space births are rooted in maternal and child health concerns, improved when pregnancies are sufficiently spaced. Some mothers caring for young infants reported these health concerns as an additional motivation to end their pregnancy.

With a baby of only four months, and you are pregnant again, at that point if you don’t have support or if God doesn’t help you, you could suffer twice as much, that is, in addition to caring for the baby, you have to take care of the pregnancy, and that’s difficult there, you can’t be in this situation and be in good health, it’s complicated.

(20 years old, 1 child, primary level education, living in a rural area at time of abortion).

Beyond stigma and health, economic considerations were the most prominent motivations to terminate a pregnancy among young couples struggling to make ends meet while raising a young family. This was the case of one woman who explained that when she became pregnant, her baby was only seven months old, and she quickly realized that her spouse’s situation as an intern (a trainee) would not provide sufficient income to support an additional child so soon.

The baby is still small, there will be a feeding issue, and with the pregnancy, one can also develop diseases, and all that, that’s more expenses that add up while he cannot even manage to provide for the current expenses; so, we had to do it like that since it’s really complicated.

(23 years old, 1 child, secondary level education, living in an urban area at time of abortion).

#### Care-seeking

The partner’s involvement in decisions to terminate the pregnancy meant that they also supported women’s access to care, serving as a primary source of information and an economic resource to cover costs. This is reflected in one respondent’s story (20 years old, 1 child, primary level education, living in an urban area at time of abortion), as her husband drove her to a place that looked like a health center to see a “retired doctor”. She joined a line of pregnant women who also seemed to be there for abortions. She underwent surgery following an injection, with no further complications after taking pills for a few days at home. Her husband paid 10,000 CFA (US$16) for the abortion. Partners also served as advocates for women in healthcare facilities, as in the case of another married respondent (32 years old, 2 children, primary level education, living in a rural area at time of abortion), who was initially rejected by the community health care center but ultimately received misoprostol after her husband (absent due to work) convinced the provider on the phone. She paid 10,000 CFA (US$16) for the pills.

While most partners supported women’s care decisions, they had little influence on the use of recommended (n = 3/5) or non-recommended (n = 2/5) methods to induce the abortions. Only one woman experienced complications, suggesting safer procedures than those obtained by the adolescents.

### Third typology: Abortion to avoid childbearing among single mothers

Five single mothers between the ages of 21 and 42, who all had children from previous relationships but were mostly living with their family, comprised the last type identified in this study.

#### Context of pregnancy

These single mothers became pregnant in the context of a casual or short-term relationship. While sexually experienced, these women were in unstable relations described as “one-night stands” or “dead-end” relationships, and had not anticipated sexual relations, which were therefore unprotected.

#### Abortion decision

Similar to prior groups, social stigma and economic constraints were important motivations for ending the pregnancy, although these women also acknowledged lack of partner support as a primary reason.

Failed relations were a common theme in this group of single mothers. In some instances, women broke up with their partners as men were engaged in other relations and were unwilling to take paternity responsibility. This was the case for one respondent (21 years old, 1 child, primary level education, living in an urban area at time of abortion) who discovered her boyfriend was already married at the time she became pregnant. While she would have liked to continue the pregnancy, her partner refused to recognize the pregnancy and asked her to abort, a decision her mother supported to avoid family “dishonor.”

The repetition of out-of-wedlock birth was a source of additional social stigma for these single mothers who had already “failed” previous relationships and were raising their children alone. This was the case of another respondent (24 years old, 1 child, primary level education, living in an urban area at time of abortion) who was living with her siblings when she became pregnant and eventually decided to have an abortion because she would be ostracized by her family for repeating the same “mistakes.” A third respondent (32 years old, 4 children, primary level education, living in an urban area at time of abortion), who already had three children from different relationships, also felt she had no choice but to terminate the pregnancy in order to save her image.

I waited for a week and my periods were not coming, so I told him my periods were not coming although I was not married yet […] but I already had SX [child initials] without being married and it wasn’t going very well because I was always home. So, if I was again going to have a baby right away with SX, people were going to talk, and my girlfriend told me she would help me and she went to buy the product (...)

(24 years old, 1 child, primary level education, living in an urban area at time of abortion).

What led me to get an abortion, my first daughter has a different father, my second son has a different father, my two children they have the same father. What would you do if you were in my shoes?

(32 years old, 4 children, primary level education, living in an urban area at time of abortion).

(long silence) In fact, I did not want to remove the pregnancy but when my mom learned I was pregnant, she told me to get an abortion, especially that the author [her partner] of the pregnancy refused to take responsibility, so she told me to get an abortion or to get my things and leave her house, which is why I called the author of the pregnancy and we went to the clinic to remove it.

(21 years old, 1 child, primary level education, living in an urban area at time of abortion).

Overall, the women in this typology had some autonomy in their abortion decision-making, particularly because they were more likely to make the decision independently from a partner, but still were subject to social and familial pressures.

#### Care-seeking

Given the conflicting relationships with partners, these single mothers mostly sought support from friends, sometimes relying on their partner’s financial resources to pay the cost. One respondent was an exception, as she was accompanied by her partner to the “clinic” where the “doctor” prescribed misoprostol after having given her an injection. She was five months pregnant at the time and started bleeding the same day, but she had no complications after the expulsion of the fetus.

All other women relied on their personal network, either friends or family, who recommended a diversity of sources including informal providers. For example, one respondent (29 years old, 3 children, primary level education, living in an urban area at time of abortion) could not reach her partner, so she turned to her friend to ask for help. Her friend recommended a traditional practitioner who had successfully provided an abortion for an acquaintance. She was given two black balls to insert in the vagina, and she paid 5000 CFA (US$8). She experienced heavy bleeding and excruciating pain for several days, which led her to the hospital for curettage. On the other hand, another respondent (42 years old, 5 children, secondary level education, living in an urban area at time of abortion) contacted her sister who had had an abortion and set up a home visit with the “nurse” who had performed her abortion. She got an initial injection with no effect, followed by misoprostol. She bled for 3 days and expelled the fetus one month later. She paid the provider a total of 20,000 CFA (US$32), including 5000 CFA (US$8) paid by her partner.

Two women were considered outliers who did not fit within one of the three types: one woman was coerced into having an abortion by her employer with whom she lived, and the other woman terminated her pregnancy without knowing when her husband gave her abortion pills, without her consent, under the guise that the pills were headache medication.

## Discussion

This research draws attention to the social, economic, and legal vulnerabilities of women shaping their pathways to abortion care in Burkina Faso. Specifically, normative expectations about childbearing and gender dynamics shape women’s pregnancy and abortion care decisions that revolve around secrecy and social capital in a restrained social and legal environment.

In all cases, women became pregnant following unprotected intercourse, often because they lacked information about pregnancy risk and protection. Lack of knowledge was particularly prevalent among girls starting their sexual careers, in line with 2020 survey results showing that only half of adolescents and young women ages 20–24 had satisfied demand for contraception, and 15% of adolescents and 33% of young women ages 20–24 years had discussed family planning with a health care worker or a provider in a facility in the last year [30]. These results call for sustained investment in adolescent and youth sexual and reproductive health (AYSRH) in Burkina Faso, which has, with the support of UNPFA, introduced comprehensive sexual education in its national teacher training curriculum in 2019 [31], but had not fully implemented the program in schools, due to social norms about adolescent sexuality and competing educational priorities. In addition, sexual education efforts need to expand beyond schools to address widespread AYSRH inequities by education in the region [32].

Strong cultural norms about sexuality and childbearing determined normative expectations about sex, marriage, and childbearing across the life course [33]. These social norms condemn sexual relations and childbearing before marriage but strongly tie women’s social status to having children as soon as they get married. These norms resonate in young unmarried women’s decisions to terminate a pregnancy, out of fear of social rejection by their families. These findings are also reported in a qualitative study in Kenya and India [34], but extend to older single mothers who are seen as repeatedly “breaking the rules’’ by giving birth out of wedlock. Within the context of marriage, traditional norms prescribe sexual abstinence in the postpartum period to ensure child spacing although these practices have relaxed in recent years, with earlier resumption of sexual activity leading to shorter pregnancy intervals that challenge normative values. These normative values weighed in married couples’ decisions to terminate a pregnancy that was too soon after a recent birth, although economic constraints also informed their decisions.

Beyond stigma, partnership dynamics were prominent forces shaping women’s abortion experiences. Reproductive coercion was a common experience, with some male partners forcing women to terminate their pregnancy because they refused to take on paternity responsibilities, while others expected women to continue an unwanted pregnancy which led to covert pregnancy terminations. On the other hand, partners were a major source of support for abortion care, providing financial resources and activating their social network to facilitate access to care. These results are in line with findings from qualitative studies in urban Zambia and Kenya, highlighting the positive and negative influences of male partners on abortion trajectories [18, 35]. In our study, the supportive role of married partners seemed to open the doors to facility-based abortions that were associated with fewer complications, while the limited social support of unmarried partners, who were either non-involved or only financially implicated, led to more diverse trajectories and more frequent complications.

In the absence of partner support, women relied on their friends for advice, and often turned to non-recommended abortion means due to financial constraints. Friends’ recommendations were often born out of their or their acquaintances’ experiences, rather than evidence-based information. Many were directed towards clandestine providers, including traditional healers, or were advised to take pills with no further instructions, failing to meet the World Health Organization’s (WHO’s) standards for safe self-managed abortion [22]. These results echo Osur and colleagues’ qualitative study in Kenya highlighting the important role of social networks in informing pathways to clandestine unsafe abortion in Kenya [36]. The lack of partner support also resulted in financial constraints, leaving women with no other affordable option than seeking help from traditional healers or from clandestine drug vendors, even if they perceived them as potentially dangerous. These results are on par with population-based studies in the region showing poor women are more likely to seek abortion care from non-clinical sources providing non-recommended methods [13, 14], associated with potentially higher morbidity and mortality.

Many women in our study described abortion complications, some leading to hospitalization. While younger women (mainly undergoing clandestine abortions) seemed particularly affected, complications were widespread, even when women sought care from a clinical source. In addition, many women who self-managed their abortion with medication were provided little information about how to manage their symptoms or what to expect, leaving them with many uncertainties surrounding the process. In our study, complications resulted from the intersection of pregnancy conditions and quality of care. Many women described late pregnancy terminations, as they did not recognize the initial signs of pregnancy and/or experienced delays in accessing effective care, either because of provider refusal to provide safe abortions or because they initiated care with non-recommended methods. The frequency of abortion complications following clinical care also raises concerns about the training of providers, especially those offering this service clandestinely. With the expansion of medication abortion, within and outside of the healthcare sector, there is an urgent need to expand provider training and diffuse evidence-based information to not only allow mid-level providers to deliver care in primary care settings but also support community health workers in delivering information about postabortion care to improve women’s health.

While this study complements our understanding of abortion care experiences in the context of Burkina Faso’s commitment to expand family planning services and reduce unsafe abortion mortality and morbidity, our findings need to be interpreted with several limitations in mind. First, the selection of women in the study was conditioned on their willingness to report and share their abortion experiences. Other findings from this study suggest substantial respondent underreporting, with one year induced abortion incidence estimates from direct reports being one sixth as high as indirect estimates [37]. This may affect our qualitative results if underreporting of abortion is related to abortion care decisions, though we observed similar abortion experiences including methods, sources of care, and safety, through direct and indirect approaches limiting our concerns [37]. Second, some participants felt uncomfortable when sharing their abortion experience. To build trust, interviewers were trained to conduct interviews on sensitive topics and underwent a values clarification training to acknowledge their own feelings about abortion. In addition, most interviewers had built rapport with participants prior to the qualitative study when conducting a quantitative PMA survey interview. Nevertheless, the sensitivity of the topic may have limited women’s willingness to share their whole experience to reduce stigma-related anxiety.

Despite these limitations, this study provides new insights on abortion experiences in Burkina Faso, showing how abortion care is conditioned on social norms, gender power dynamics, and economic resources, contributing to the growing body of literature on women’s abortion trajectories in the country [3, 4, 10, 16, 17, 28]. In this patriarchal society, partners are instrumental in women’s abortion experiences, from coercing decisions to supporting healthcare access [17]. Beyond partner implication, social stigma and lack of economic resources amplify barriers to evidence-based information and care in this legally restricted abortion environment.

## Conclusion

This research suggests that legal restrictions on abortion fuel an informal system of care where non-clinical sources and non-recommended methods predominate. When information is scarce and highly stigmatized, women turn to their social network, including partners and friends for advice, with significant implications for their care.

As Burkina Faso formalizes its framework for AYSRH education for youth and adolescents, comprehensive sexual education coupled with community-based interventions to promote gender equitable relationships and address social stigma related to women’s sexuality could reduce reproductive coercion and unintended pregnancies. To reduce complications of unintended pregnancies, legal and health systems reforms are also needed to expand the legal conditions for abortion and improve quality services for contraception, abortion, and postabortion care through task-shifting in primary care. Policies also need to adapt to the reality of self-managed abortion.

## Data Availability

The datasets generated and/or analyzed during the current study are not publicly available due to the qualitative nature of the data and identifying personal and geographic information but are available from the corresponding author on reasonable request.

## Abbreviations

AYSRH: Adolescent and youth sexual and reproductive health
BSPH: Bloomberg School of Public Health
IDI: In-depth interview
ISSP: Institut Supérieur des Sciences de la Population
PMA: Performance monitoring for action
WRA: Women of reproductive age
